# Supplementary material for: A New Methodological Approach Integrating Motion Capture and Pressure-Sensitive Gait Data to Assess Functional Mobility in Parkinson’s Disease: A Two-Phase Study
Source: Sensors (Basel). 2025 Sep 29;25(19):5999. doi: 10.3390/s25195999 (PMC12526586; doi:10.3390/s25195999)
Supplement: Supplementary file 1 [file sensors-25-05999-s001.zip › sensors-3853177-Supplementary.pdf]

Instruction Manual

Performance Score Functional Mobility Assessment in Parkinson's (FMA-P)

A new measure has been developed to quantify and describe the specific tasks of the FMA-P. This is a list of seven tasks (10 items in total, plus four descriptions) assigned to the tasks of the FMA-P. A four-point ordinal scale (0 indicates highest level of function; 3 indicates lowest level of function) is used to assess the quality of performance with a maximum total score of 30. The FMA-P is assessed three times in series and the average score from across each repetition has to be calculated for each task.

The participants must perform the FMA-P task as quickly as possible on a five meter long gait mat (a three metre walk is possible before the turn). The data is recorded using motion capture system and video recordings.

The FMA-P sequence is composed of A. Sitting to standing and standing to sitting, B. Walking forward , C. Walking through a visual obstacle (doorway), D. Turning (180 º), E. Bending to pick up a Key from the ground, and grasp it, F. Placing the Key at the height of the shoulder.

Figure S1. *Diagram of the Functional Mobility Assessment in Parkinson's (FMA-P) sequence*

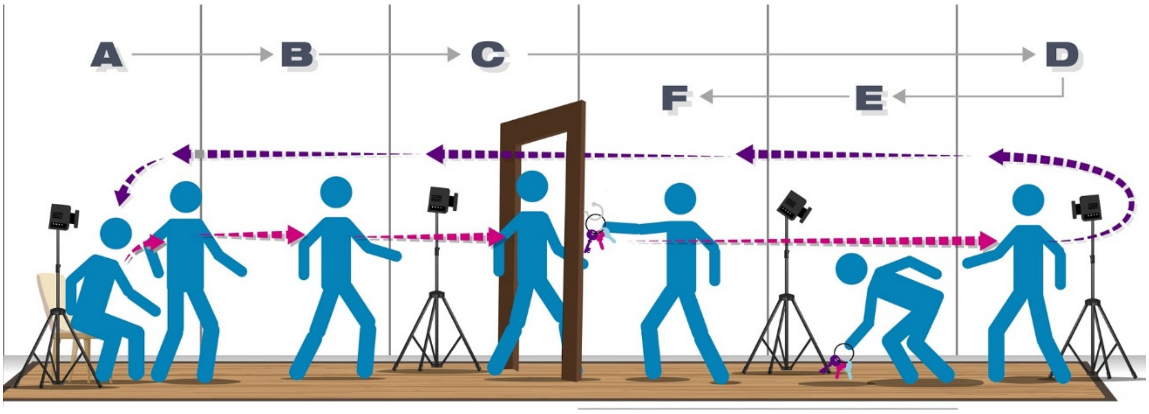

Note: The stool should have no backrest to enable good camera visibility and must be stable (i.e., not a swivel chair) to ensure the safety of the participants. The chair must be adjusted to the correct height so that a 90-degree angle is achieved at the knees.
The hook for hanging the key must be positioned at shoulder height.
The doorway is included to assess "freezing" of gait in Person's with Parkison's (PwP). Freezing is one of the most disabling symptoms of locomotion, as the feet involuntarily "stick" to the floor, leading to a higher risk of falling[1,2]. Previous studies have shown that freezing can be triggered by narrow spaces or doorways [3,4].

Instructions in how to analyse the data with Motion Capture Video-Analysis

Tasks Detailed

1. SITTING TO STANDING

Postural stability

( ) 0 able to rise without using hands and stabilise independently
( ) 1 able to rise with arm swing
( ) 2 able to rise using hands for support
( ) 3 able to rise after more than one try

Note: To assess whether arm swing is used to assist standing up, the wrist marker and sternum marker are analysed. Arm swing support is indicated as soon as the wrist markers are lifted at the level of the sternum during standing up from the chair.

Descriptive

( ) single foot offset	( ) double foot offset
( ) hand/s on chair	( ) hand/s on knee/thigh
( ) arm swing assist	( ) other:

Note: If a single foot offset or only one hand support was observed, it should always be indicated which side (left or right foot/hand) was required as a support.

Balance once standing

( ) 0 able to stand safely and upright
( ) 1 able to stand safely with bending of the back and with 1-2 steps till upright
( ) 2 able to stand safely with bending of the back and with >2 steps till upright
( ) 3 able to stand with postural instability and/or remain standing before locomotion

Note: The sternum marker and the cervical vertebra 7 (C7) are used to assess the erectness of the upper body (standing upright). The first step is counted as soon as the second foot leaves the ground after the heel-strike of the first foot (toe-off).

Figure S2. *Toe-off and Heel-strike during walking.*

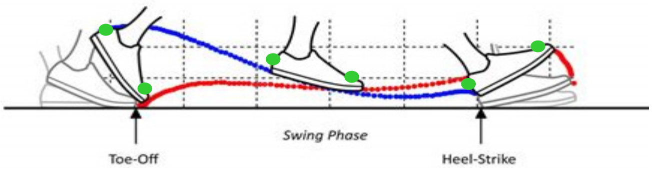

( ) 0 able to walk with upright posture and normal shoulder and pelvic rotation
( ) 1 able to walk upright with less normal shoulder and pelvic rotation
( ) 2 able to walk with a stooped posture, less shoulder and pelvic rotation
( ) 3 able to walk with poor postural balance

Note: Less shoulder and pelvic rotation are often accompanied by less knee flexion during walking.

Descriptive

( ) symmetrical arm swing with good swing amplitude	( ) arm swing asymmetric (note which side)
( ) symmetrical arm swing with less amplitude	( ) arm swing from the shoulder with 90° flexed elbow
( ) arm swing from the elbow only	( ) other:

Note: An asymmetrical arm swing is assessed in the sagittal plane, i.e. an asymmetrical arm swing amplitude or an uncoordinated arm swing. It should be noted which arm has a lower amplitude or does not swing simultaneously.

Walking stability

( ) 0 able to walk straight and steady
( ) 1 able to walk straight but with a lack of proper rolling/heel strike
( ) 2 able to walk straight but with small steps and a lack of proper rolling/heel strike
( ) 3 able to walk with shuffling steps and/or sudden gait acceleration

Note: A non-proper heel strike can be characterised by a smaller toe-off or heel-strike angle (less than 45 degrees), or a lower foot height, usually accompanied by a lower knee flexion [5]. An improper heel-strike can also be characterised by slumping of the forefoot.

3. WALKING THROUGH A VISUAL OBSTACLE

Continuous walking speed

( ) 0 able to walk through the doorway straight and continuous
( ) 1 able to walk through the doorway slowly and with small steps
( ) 2 able to walk through the doorway with a short “freezing” episode
( ) 3 able to walk through the doorway with a “freezing” episode and shuffling

Note: This task is always evaluated in relation to the entire locomotion, e.g. the velocity is reduced compared to walking without an obstacle when passing the doorway [3].

4. TURNING

Postural stability

( ) 0 able to turn fluently
( ) 1 able to turn but not fluently/on block (no extra steps)
( ) 2 able to turn but not fluently/on block with small/extra steps and <= 4 steps
( ) 3 able to turn not fluently/on block with small/extra steps and > 4 steps

Note: Due to loss of postural control, PwP tend to use small steps with adjustments of the feet to maintain balance when changing direction [6] . It has been demonstrated that PwP rotate the head, thorax and pelvis simultaneously (turning on block), while healthy adults perform a predominant cranial to caudal rotation[7].
The number of steps from the first heel strike of the turning foot to the first heel strike of the swing leg after the turn is calculated as the number of steps.

5. PICK UP, GRASP, PLACE AN OBJECT

Postural stability

( ) 0 able to bend and rise up safely and easily with continuous locomotion
( ) 1 able to bend and rise up with >1 step and/or obviously remain standing between continuous locomotion
( ) 2 able to bend and rise up with postural imbalance but with support from placing hand on knee/thigh
( ) 3 able to bend and rise up but with fall risk (stumble)

Note: In the case of postural instability, an additional step is often added for stabilisation, or the movement sequence when picking up the key is supported by stabilisation with one hand on the knee.

Functional reach

( ) 0 able to grab and pick up safely and easily (one try)
( ) 1 able to grab and pick up but slowly
( ) 2 able to grab and pick up with difficulty (not fluently/extra step/s)
( ) 3 able to grab and pick up with more than one try (key dropped)

Note: A non-fluently movement performance means that there is a pause in the movement sequence or that the speed of the movement execution changes during the task, often accompanied by extra steps for stabilisation.

Descriptive

( ) lunge to reach	( ) stop and stoop
( ) extra step	( ) stop and knee bend
( ) hand on knee support	( ) other:

Note: **"**Stop and stoop" means that the participant does not bend their knees (squat) or only bends their knees slightly to pick up the key.

Functional placing

( ) 0 place the object safely and easily (continuous fluid motion)
( ) 1 place the object slowly (stands obviously still and place/hold feet parallel)
( ) 2 place the object with difficulty (not fluently, needs extra step/s)
( ) 3 place the object after more than one try

Note: Less manual control is often judged by the fact that it takes more time to place the key, i.e. obviously standing still, indicating less postural balance, or having less dexterity to place the Key (not fluently)[8].

6. STANDING TO SITTING

Postural stability

( ) 0 sits safely and fluidly
( ) 1 controls descent with obvious bend from trunk
( ) 2 controls descent by using 1 or 2 hands with obvious bend from trunk
( ) 3 sits independently but has uncontrolled descent and support with hands to control

the descent

Note: Postural imbalance in PwP can be characterised by a longer and obvious trunk bending phase when sitting down[9]. This is assessed with a higher flexion of the hips (>= 90 ^{o}) when sitting down.

Descriptive

( ) hand on knee/thigh	( ) hands on chair
( ) two hand support	( ) one hand support
( ) slump	( ) other:

Note: If only one hand support was observed, it should always be indicated which side (left or right hand) was required as a support.

References:

1. Mancini M, Bloem BR, Horak FB, Lewis SJG, Nieuwboer A, Nonnekes J. Clinical and methodological challenges for assessing freezing of gait: Future perspectives. Movement Disorders. 2019;34(6):783-790. doi:10.1002/mds.27709
2. Rahimpour S, Gaztanaga W, Yadav AP, et al. Freezing of Gait in Parkinson's Disease: Invasive and Noninvasive Neuromodulation. Neuromodulation. 2021;24(5):829-842. doi:10.1111/ner.13347
3. Cowie D, Limousin P, Peters A, Hariz M, Day BL. Doorway-provoked freezing of gait in Parkinson's disease. Movement Disorders. 2012;27(4):492-499. doi:10.1002/mds.23990
4. Cowie D, Limousin P, Peters A, Day BL. Insights into the neural control of locomotion from walking through doorways in Parkinson's disease. Neuropsychologia. 2010;48(9):2750-2757. doi:10.1016/j.neuropsychologia.2010.05.022
5. Shin KJ, Park J, Ha S, et al. Decreased foot height may be a subclinical shuffling gait in early stage of Parkinson's disease: A study of three-dimensional motion analysis. Gait & Posture. 2020;76:64-67. doi:10.1016/j.gaitpost.2019.11.005
6. Stack, E.; Ashburn, A. Fall Events Described by People with Parkinson's Disease: Implications for Clinical Interviewing and the Research Agenda. Physiother. Res. Int. 1999, 4, 190–200. https://doi.org/10.1002/pri.165.
7. Yang WC, Hsu WL, Wu RM, Lu TW, Lin KH. Motion analysis of axial rotation and gait stability during turning in people with Parkinson's disease. Gait & Posture. 2016;44:83-88. doi:10.1016/j.gaitpost.2015.10.023
8. Johnels, B.; Ingvarsson, P.E.; Thorselius, M.; Valls, M.; Steg, G. Disability Profiles and Objective Quantitative Assessment in Parkinson's Disease. Acta Neurol. Scand. 1989, 79, 227–238. https://doi.org/10.1111/j.1600-0404.1989.tb03743.x.
9. Inkster, L.M.; Eng, J.J. Postural Control during a Sit-to-Stand Task in Individuals with Mild Parkinson's Disease. Exp. Brain Res. 2004, 154, 33–38. https://doi.org/10.1007/s00221-003-1629-8.
